# Supplementary material for: The role of microstructure in the thermal fatigue of solder joints
Source: Nat Commun. 2024 May 20;15:4258. doi: 10.1038/s41467-024-48532-6 (PMC11106336; doi:10.1038/s41467-024-48532-6)
Supplement: Supplementary file 1 — Supplementary Information [file 41467_2024_48532_MOESM1_ESM.pdf]

Supplementary Information

The role of microstructure in the thermal fatigue of solder joints

J.W. Xian[1,2*], Y.L. Xu[1,3*], S. Stoyanov[4], R.J. Coyle[5], F.P.E. Dunne[1], C.M. Gourlay[1*]

[1]Department of Materials, Imperial College London, SW7 2AZ, London, United Kingdom
[2]School of Materials Science and Engineering, Dalian University of Technology, Dalian 116024, China
*[3]Institute of High Performance Computing (IHPC), Agency for Science, Technology and Research (A*STAR), 1 Fusionopolis Way, #16-16 Connexis, Singapore 138632, Republic of Singapore*
[4]School of Computing and Mathematical Sciences, University of Greenwich, SE10 9LS, London, United Kingdom
[5]Nokia Bell Labs, Murray Hill, New Jersey, USA

The frequency of occurrence of β-Sn microstructure types:

Supplementary Table 1 β-Sn microstructure types in the 84CTBGA packages at time zero (as soldered) and after 7580 thermal cycles from 0/100 °C. Different packages were analysed at time zero and after thermal cycling.

	Number of joints	Single grain	Multigrain		
			single ring	double ring	independent
Time zero	20	5 (25%)	13 (65%)	2 (10%)	0 (0%)
Thermally cycled	84	16 (19%)	50 (60%)	15 (18%)	3 (4%)

Similarity of intermetallic particle size among joints:

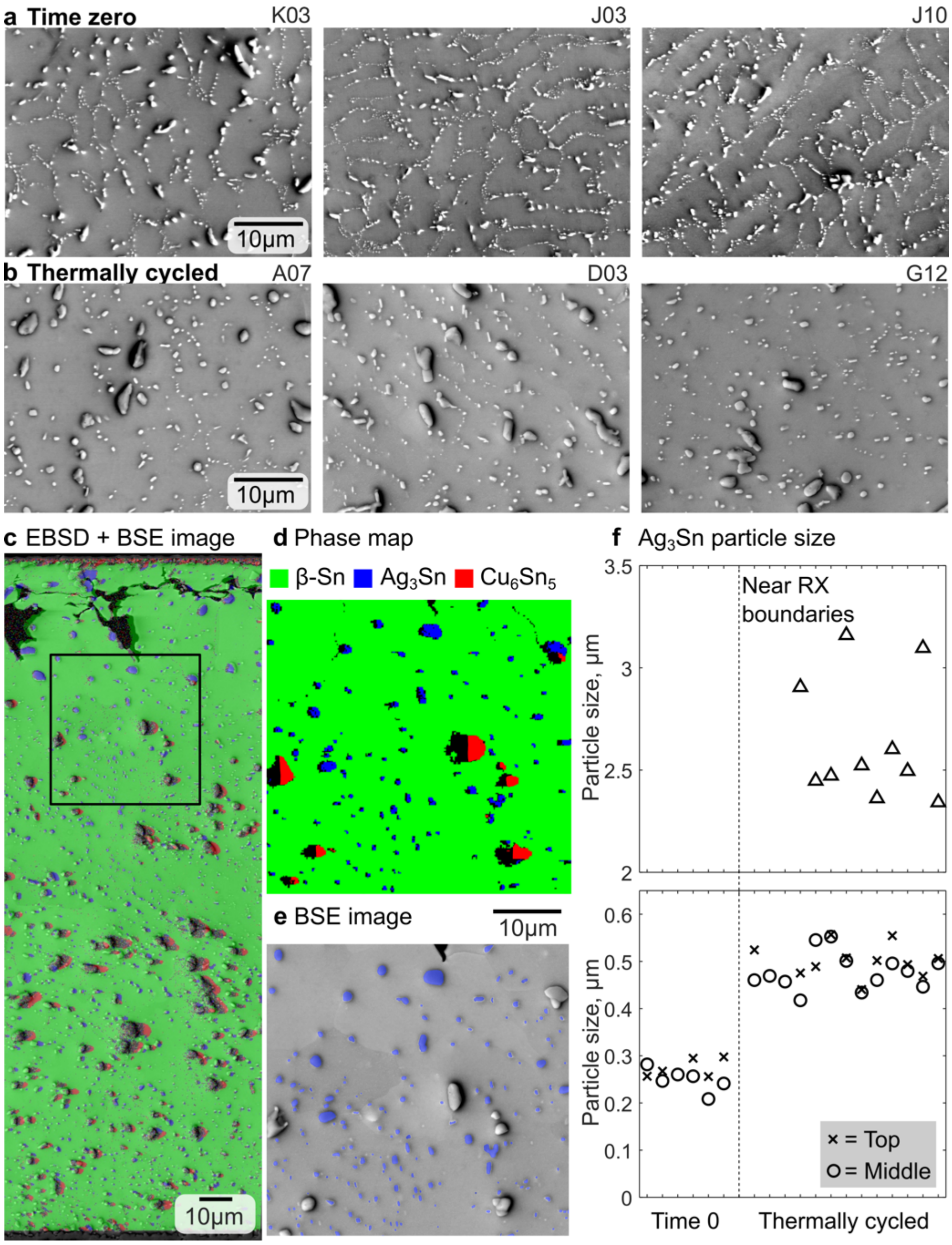

Supplementary Figure 1 Variability in eutectic Ag_3Sn particle size. a-b) BSE images taken from the middle regions of the joints in Figure 2 at time-zero and after thermal cycling respectively, c) an EBSD phase map from bottom to top of joint D03 overlayed on a BSE image, d) a cut-out from the black box, e) the BSE image with segmented Ag_3Sn particles indicated in blue. F) Ag_3Sn particle size measurements from segmented images. 6 time zero joints were measured yielding an Ag_3Sn particle size of 0.26 ± 0.03 µm (mean ± standard deviation) across both the middle (circle symbols) and top (cross symbols) locations of the solder joints. 13 thermally cycled joints were measured yielding a Ag_3Sn particle size of 0.49 ± 0.04 µm in the middle and top regions away from the localised strain/damage. Triangle symbols indicate the mean size of Ag_3Sn in recrystallised regions (See Fig. 4 in the main paper) in the same thermally cycled joints. Compared with the strong variability in tin grain structure reported in the main paper, the Ag_3Sn particle size is similar among time zero joints and similar among thermally cycled joints.

Comparison of macro-scale models: Chaboche+Garofalo model vs. Anand model:

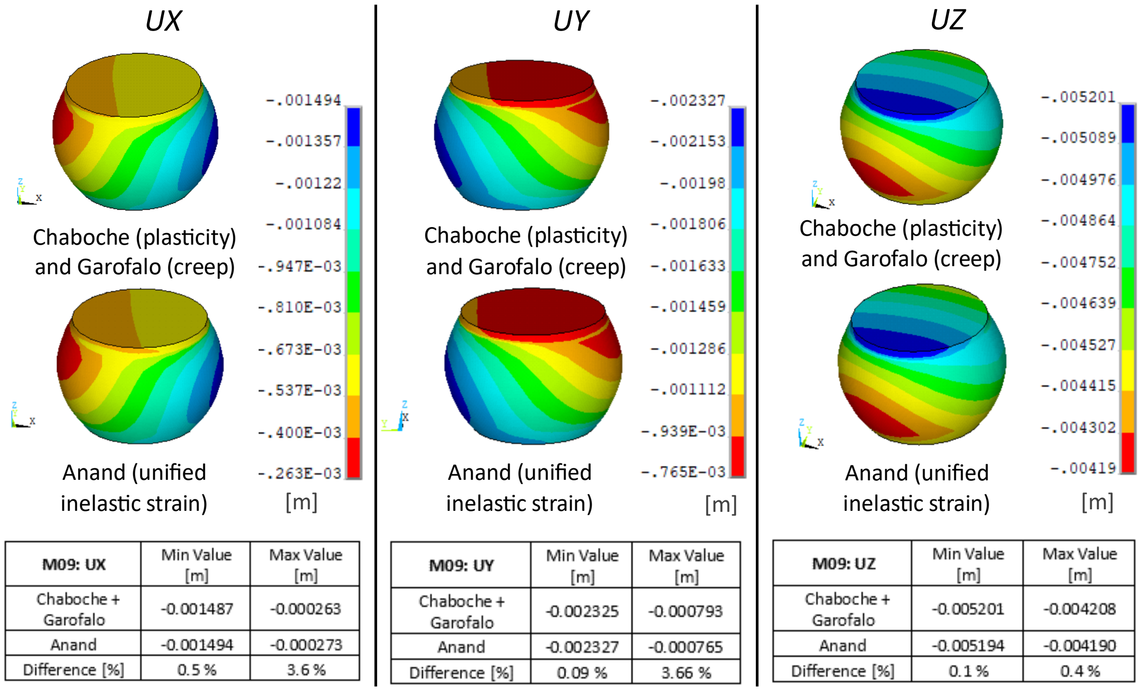

**M09: UX**	Min Value [m]	Max Value [m]
Chaboche + Garofalo	-0.001487	-0.000263
Anand	-0.001494	-0.000273
Difference [%]	0.5 %	3.6 %

**M09: UY**	Min Value [m]	Max Value [m]
Chaboche + Garofalo	-0.002325	-0.000793
Anand	-0.002327	-0.000765
Difference [%]	0.09 %	3.66 %

**M09: UZ**	Min Value [m]	Max Value [m]
Chaboche + Garofalo	-0.005201	-0.004208
Anand	-0.005194	-0.004190
Difference [%]	0.1 %	0.4 %

Supplementary Figure 2 Solder joint M09 contour plots of displacement (*UX*, *UY* and *UZ*) at the high-temperature extreme of the thermal cycle (100°C) predicted under two different setups for the Sn-3.0Ag-0.5Cu material behaviour: (1) Chaboche (kinematic hardening rule) and Garofalo (creep), and (2) Anand viscoplastic model. Peak values of displacements and their percentage differences with the two model setups are tabulated under the contour plots.

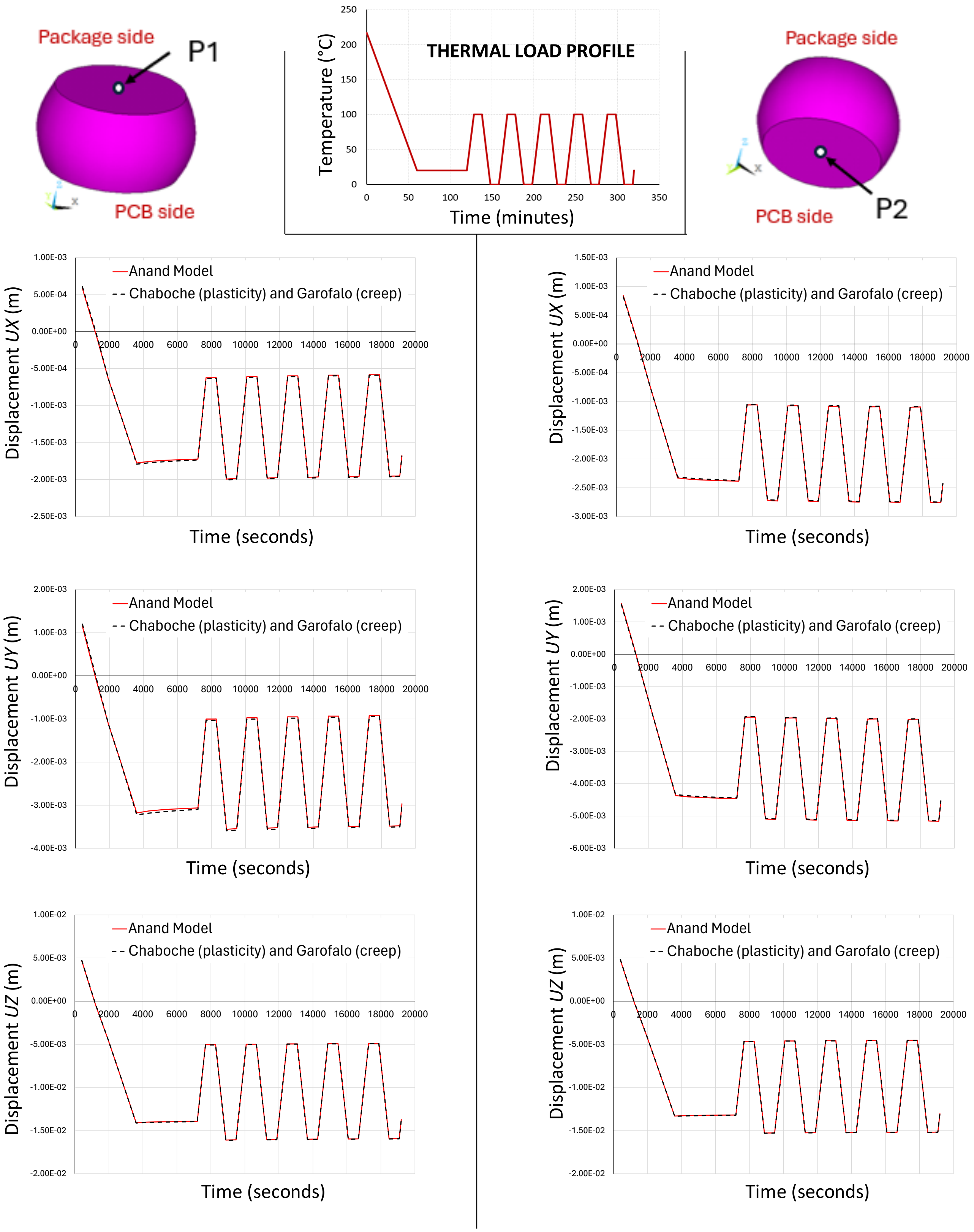

Supplementary Figure 3 Transient displacement results (*UX*, *UY* and *UZ*) of the central point at the top interface (P1) and bottom interface (P2) of solder joint M9, predicted under two different setups for the Sn-3.0Ag-0.5Cu material behaviour: (1) Chaboche (kinematic hardening rule) and Garofalo (creep), and (2) Anand viscoplastic model. The top centre graph details the profile of the simulated isothermal load, representing the post-reflow cooldown of the assembly (causing residual stress) followed by five temperature cycles of 0-100°C.

Parameters used in macro-scale and micro-scale models:

Supplementary Table 2 Chaboche kinematic hardening model parameters for Sn-3.0Ag-0.5Cu

Temp [°C]	σ_0 [MPa]	C_1	γ_1	C_2	γ_2	C_2	γ_2
-40	36.46	49594	5200	24330	1485	984	211
25	24.96	27809	5162	13481	1476	548	211
75	19.05	18714	5140	9185	1473	376	210
125	14.64	12915	5145	6502	1474	268	210

Supplementary Table 3 Garofalo-Arrhenius creep strain rate model parameters for Sn-3.0Ag-0.5Cu

Temp [°C]	A [s^{-1}]	B [MPa^{-1}]	n [−]	Q/R [K^{-1}]
25	31974	0.02447	6.41	6498.32
75	79512			
100	134720			
125	213760			

Supplementary Table 4 Key properties used in the CPFE model. $\bar{r}_{IMC}$ is the mean radius, 0.21μm in [1], of intermetallic particles embedded within β-Sn in Sn-3Ag-0.5Cu.

Property	Unit	Symbol	Value [T in °C]	Ref.
CTE	[$°C^{-1}$]	$a_{11}= a_{22}$	$14.195\times10^{-6} + 25.53\times10^{-9}\times T$	[2]
		a_{33}	$28.662\times10^{-6} + 56.79\times10^{-9}\times T$	
Elasticity	[GPa]	$E_{11}=E_{22}$	$25.3 - 0.083 \times T$	[3]
		E_{33}	$68.3 - 0.072 \times T$	
		G_{12}	$24.4 - 0.020 \times T$	
		G_{13}	$22.5 - 0.026 \times T$	
	[-]	ν_{12}	0.46	
		ν_{13}	0.22	
Plasticity	[MPa]	$\tau_{c0}^{\langle 001\rangle}$	$4.9 - 0.02 \times T$	
	[μm^{-2}]	λ_1	$-260 \times \bar{r}_{IMC} + 1215$	
	[$\mu m^{-2}s^{-1}$]	λ_2	$-4.7 \times \bar{r}_{IMC} + 15.2$	
	Multiplicity	Slip system	CRSS ratio	
	2	{100}<001>	1	[4]
	2	{110}<001>	1	
	2	{100}<010>	1.05	
	4	{110}<1-11>/2	1.1	
	2	{110}<1-10>	1.2	
	4	{100}<011>	1.25	
	2	{001}<010>	1.3	
	2	{001}<110>	1.4	
	4	{011}<01-1>	1.5	
	8	{211}<01-1>	1.5	

Supplementary references:

[1] Xu, Y. *et al.* Intermetallic size and morphology effects on creep rate of Sn-3Ag-0.5Cu solder. *International Journal of Plasticity* **137**, 102904, (2021).

[2] Xian, J. W. *et al.* Anisotropic thermal expansion of Ni_3Sn_4, Ag_3Sn, Cu_3Sn, Cu_6Sn_5 and βSn. *Intermetallics* **91**, 50-64, (2017).

[3] Xu, Y. L. *et al.* Multi-scale plasticity homogenization of Sn-3Ag-0.5Cu: From β-Sn micropillars to polycrystals with intermetallics. *Materials Science and Engineering: A* **855**, 143876, (2022).

[4] Zamiri, A., Bieler, T. R. & Pourboghrat, F. Anisotropic crystal plasticity finite element modeling of the effect of crystal orientation and solder joint geometry on deformation after temperature change. *Journal of Electronic Materials* **38**, 231–240, (2009).
